# Identifying cross-sectoral cooperation for urban health

**DOI:** 10.1093/eurpub/ckac129.565

**Published:** 2022-10-25

**Authors:** K Matinheikki, A Liinamo

**Affiliations:** 1 School of Rehabilitation and Examination, Metropolia University of Applied Sciences, Helsinki, Finland; 2 Health Promotion, Metropolia University of Applied Sciences, Helsinki, Finland

## Abstract

Empirical evidence for collaborative health promotion practices is still limited. The Cross-Sectoral Cooperation Self-Assessment Tool (CroCo SA) presented here provides a tool for cities to assess their current commitment to promote urban health and well-being across sectors. The SA tool was validated by co-operating with 9 pilot cities and seven countries in the framework of the Healthy Boost project funded by Interreg Baltic Sea Region (2019-2022). Addressing the current complexity, interdependence and emerging challenges of health promotion requires cross-sectoral cooperation and tools to assess cross-sectoral processes and make their impact visible. A digitized CroCo SA tool was developed and validated. An SA evaluation matrix was created, including 162 cells of options for reflective and guiding responses according to 6 scale x 27 items used in the SA tool. After submission of the electronic form, respondents can view anonymized results in a digital form on the Healthy Boost platform. The feedback report summarizes the results for respondents and provides, both numerical and descriptive, verbal feedback on the state of the assessed city's capacity for cross-sectoral cooperation, based on each assessment. The steps the respondents could take in their respective cities to improve cross-sectoral cooperation are outlined. Recommendations are based on the evidence found to be the prerequisites for effective cross-sectoral work for health promotion. SA can be conducted in many languages offered by the Google translation programme. Guiding feedback of CroCo SA defines areas for improvement in strategies and actions, allowing respondents also to do benchmarking and learn from other cities and countries. Self-assessment itself is a learning process.

**Key messages:**

• The CroCo SA tool offers a method for gathering information about the state of cross-sectoral cooperation for urban health promotion.

• By identifying strengths and weaknesses in cooperation, the city can build capacity for cross-sectoral cooperation.

